# Restoration of mitochondrial function by *Spirulina* polysaccharide via upregulated SOD2 in aging fibroblasts

**DOI:** 10.1016/j.isci.2023.107113

**Published:** 2023-06-14

**Authors:** Kayo Machihara, Shoma Oki, Yuka Maejima, Sou Kageyama, Ayumu Onda, Yurino Koseki, Yasuyuki Imai, Takushi Namba

**Affiliations:** 1 Research and Education Faculty, Multidisciplinary Science Cluster, Interdisciplinary Science Unit, Kochi University, Kochi 783-8505, Japan; 2 Department of Marine Resource Science, Faculty of Agriculture and Marine Science, Kochi University, Kochi 783-8502, Japan; 3 Health Care Technical G, Chiba Plants, DIC Corporation, Ichihara, Chiba 290-8585, Japan

**Keywords:** Cell biology, Cellular physiology, Functional aspects of cell biology

## Abstract

Reactive oxygen species (ROS), such as superoxide, are crucial factors involved in the stimulation of cellular aging. Mitochondria, which are important organelles responsible for various metabolic processes in cells, produce ROS. These ROS impair mitochondrial function, thereby accelerating aging-related cellular dysfunction. Herein, we demonstrated that the *Spirulina* polysaccharide complex (SPC) restores mitochondrial function and collagen production by scavenging superoxide via the upregulation of superoxide dismutase 2 (SOD2) in aging fibroblasts. We observed that SOD2 expression was linked to inflammatory pathways; however, SPC did not upregulate the expression of most inflammatory cytokines produced as a result of induction of LPS in aging fibroblasts, indicating that SPC induces SOD2 without activation of inflammatory pathways. Furthermore, SPC stimulated endoplasmic reticulum (ER) protein folding by upregulating ER chaperones expression. Thus, SPC is proposed to be an antiaging material that rejuvenates aging fibroblasts by increasing their antioxidant potential via the upregulation of SOD2.

## Highlights

• *Spirulina* polysaccharide complex (SPC) induces SOD2 expression in aging fibroblasts
• SPC restores mitochondrial function in aging fibroblasts
• SPC stimulates ER protein folding by upregulating ER chaperone expression
• SPC upregulated Nrf2, which suppressed inflammatory signaling

## Graphical abstract

**Figure:**
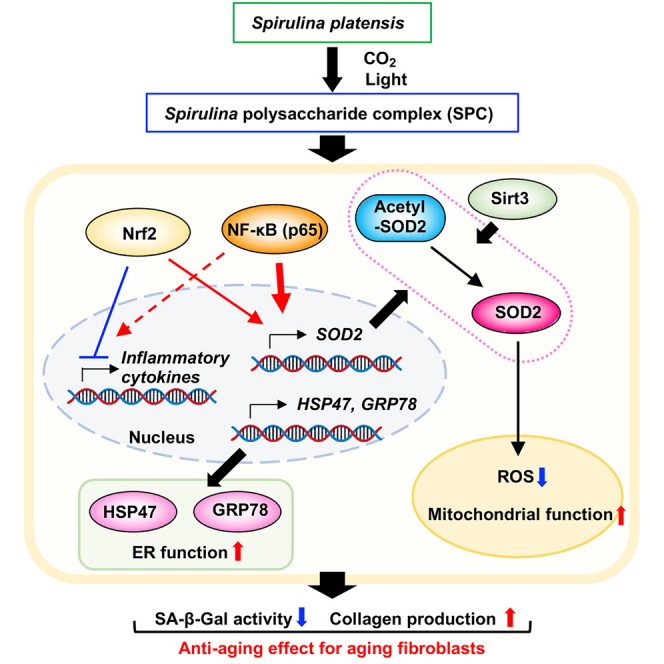

## Introduction

Aging is defined as the progressive attenuation of the biological functions of the cells of an organism.^1^ Because aging causes chronic age-related diseases and general health loss, several studies are being conducted to identify strategies to inhibit aging and thereby prolong the healthy lifespan of humans.^1^^,^^2^

Mitochondria play a crucial role in the aging process through the production of reactive oxygen species (ROS); this role is supported by the free radical theory of aging and the mitochondrial theory of aging.^3^ ROS are known to accelerate cellular senescence by damaging various cellular components, such as DNA and proteins, and inhibiting the function of various organelles.^1^ Mitochondria produce superoxide, which is the most reactive oxygen species; superoxide dismutase 1 and 2 (SOD1, localized in the cytosol; SOD2, localized in mitochondria) quickly convert superoxide to hydrogen peroxide.^4^ SOD2 is an essential antioxidant enzyme in mitochondria, and loss of SOD2 accelerates mitochondrial dysfunction and aging in cultured cells and animal models^5^^,^^6^^,^^7^; thus, SOD2 is considered an important target for mitochondrial homeostasis maintenance and antiaging. It has been proposed that aging impairs endoplasmic reticulum (ER) functions related to protein folding.^8^ ER chaperones, such as GRP78 and HSP47, play an important role in the protein folding process for extracellular matrix components like collagen and membrane proteins within the ER.^9^ Consequently, maintaining ER function is also crucial for anti-aging strategies.

Global warming, which is caused by increased carbon dioxide (CO_2_) emissions resulting from social activities of humans, has caused serious damage to the environment worldwide, including biodiversity loss, crop failure, and human diseases.^10^ Thus, sustainable practices aimed at reducing the atmospheric CO_2_ concentration are needed for humans to live a long and healthy life. Several methods have been developed to reduce atmospheric CO_2_, such as the use of microalgae to convert atmospheric CO_2_ to biomass, such as foods, fuel, and drugs, through photosynthesis.^11^ Microalgae perform biofixation of CO_2_ with high efficiency using large areas, which are difficult to use for agriculture.

*Spirulina platensis* is a filamentous and photosynthetic cyanobacterium that has been used to produce biofuel and nutrient supplements.^12^^,^^13^ *S. platensis* polysaccharides are known to have various biological activities, such as anticancer, antibacterial, and antioxidant activities. It has been reported that *Spirulina* polysaccharides themselves possess antioxidant and antibacterial properties, and in terms of anticancer activity, *Spirulina* polysaccharides have been shown to activate immune cells to attack cancer cells.^14^^,^^15^^,^^16^ However, their antiaging activities have not yet been explored using an aging cell model. *Spirulina* polysaccharides have a high molecular weight, primarily consisting of glucose and galactose, with minor amounts of rhamnose, fucose, and xylose.^17^ Previous research suggests that Spirulina polysaccharides exert their effects not by entering cells, but by interacting with receptors on cell membranes, particularly in immune cells via Toll-like receptor (TLR) 2 and TLR4.^18^

In this study, we aimed to determine whether *Spirulina* polysaccharide can act as an antiaging material possessing the potential to prevent aging-related diseases. We observed that the *Spirulina* polysaccharide complex (SPC) could restore mitochondrial function by scavenging ROS and increasing collagen production through the upregulation of SOD2, which is generally downregulated in aging fibroblasts. Thus, we propose that SPC rejuvenates aging fibroblasts and is a promising antiaging material.

## Results

### Mitochondrial dysfunction is related to collagen production

**Figure 1:**
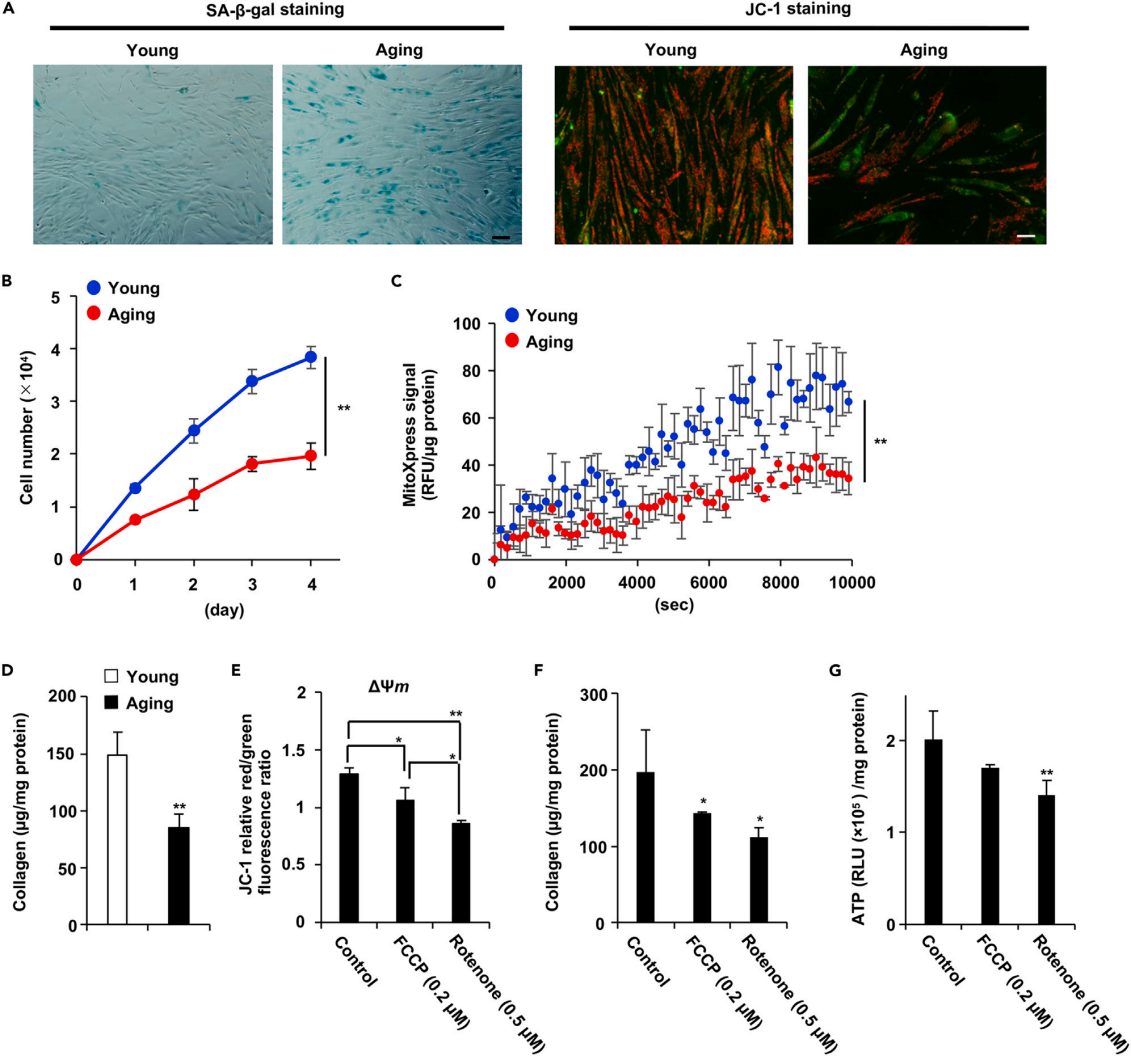
Replicative aging suppressed collagen production and mitochondrial function (A) Replicative aging decreased the mitochondrial transmembrane electric potential (ΔΨ*m*). Young and aging NB1RGB cells were stained for SA-β-gal. These images were taken at 10× magnification (scale bar, 50 μm). (left panel). The cells were incubated with JC-1 for 20 min and then subjected to fluorescence microscopy; red and green fluorescence represent JC-1 aggregates and JC-1 monomers, respectively. These images were taken at 20× magnification (scale bar, 20 μm) (right panel). (B) Aging cells exhibit a diminished growth rate compared to young cells. These cell numbers were counted by every day. (C) Mitochondrial oxygen consumption is suppressed in Aging cells. Time-resolved fluorescence (TR-F) of MitoXpress probe was converted to phosphorescence values and normalized to the cell number. Data are expressed as mean TR-F. RFU, relative fluorescence units. RFU were normalized total amount protein. (D) Replicative aging decreased collagen production. Collagen content was measured in young and aging NB1RGB cells. (E–G) Mitochondrial dysfunction suppressed ΔΨm and collagen production. Young NB1RGB cells were treated with FCCP or rotenone at the indicated concentration for 24 h. ΔΨm (E), Collagen content (F) and ATP content (G) were then measured in these cells. Collagen production and ATP levels were normalized by total amount of protein. Relative Light Unit (RLU) as a unit employed for luminescence measurements. Data are presented as the mean ± standard deviation (SD) of three simultaneously performed experiments, three wells on independent plates (B, D, F) or three wells on the same plate (C, E, G). p values were calculated using Student’s *t* test (B-D) and ANOVA following Tukey-HSD test (E-G); ∗p < 0.05, ∗∗p < 0.01.

The NB1RGB (human skin fibroblast) cell line showed increasing number of senescence associated β-galactosidase (SA-β-gal) positive cells at the proliferation and replication stages (Figure 1A, left panel). We analyzed the effect of young and aging fibroblasts on Mitochondrial membrane potential (ΔΨ*m*) using the fluorescent probe JC-1(5,5,6,6-Tetrachloro-1,1,3,3-tetraethylbenzimidazolylcarbocyanine iodide), a cationic carbocyanine dye. JC-1 aggregation occurring in the mitochondria in a potential-dependent manner can be observed as red fluorescence. Green fluorescence indicates the presence of JC-1 monomers that appear in the cytosol after mitochondrial membrane depolarization. Fluorescence microscopy revealed decreased red fluorescence in aging fibroblasts (Figure 1A, right panel). As shown Figure 1B, the cell growth rate of aging NB1RGB cells were suppressed compared to young NB1RGB cells. These data indicated that our using aging NB1RGB cells show several aging phenotypes but do not stop cell growth. We measured mitochondrial oxygen consumption levels in whole cells using an oxygen-sensitive fluorescent probe (MitoXpress) quenched by molecular oxygen via a nonchemical mechanism and found that reduced dissolved oxygen concentrations in the medium increase the probe signal.^19^ The fluorescence intensity of aging NB1RGB cells decreased in that of young NB1RGB cells, suggesting that cells consumed oxygen within the medium is decreased in aging NB1RGB cells compared to young NB1RGB cells (Figure 1C). In addition, collagen production was found to be decreased in aging fibroblasts (Figure 1D). The simultaneous occurrence of mitochondrial dysfunction and reduced collagen production in aging NB1RGB cells suggested that mitochondrial dysfunction could inhibit collagen production. We used FCCP, which decreases ΔΨ*m*, and rotenone, which inhibits mitochondrial complex I, as mitochondrial function inhibitors in young NB1RGB cells and found that these compounds reduced collagen production under suppression of ΔΨ*m* and low-ATP conditions (Figures 1E–1G). As shown in Figure 1E, Rotenone and FCCP decreased ΔΨ*m*, but Rotenone was strongly suppressed ΔΨ*m* compared to FCCP at this condition. In these results, the extent of inhibition of collagen production and ATP synthesis might have varied depending on the potency of ΔΨ*m* inhibition by FCCP and Rotenone. These results suggested that proliferative and replicative aging induced mitochondrial dysfunction, which was related to decreased collagen production.

### The hot water extract of *S.* Platensis activates mitochondrial membrane potential (ΔΨ*m*) and stimulates collagen production

**Figure 2:**
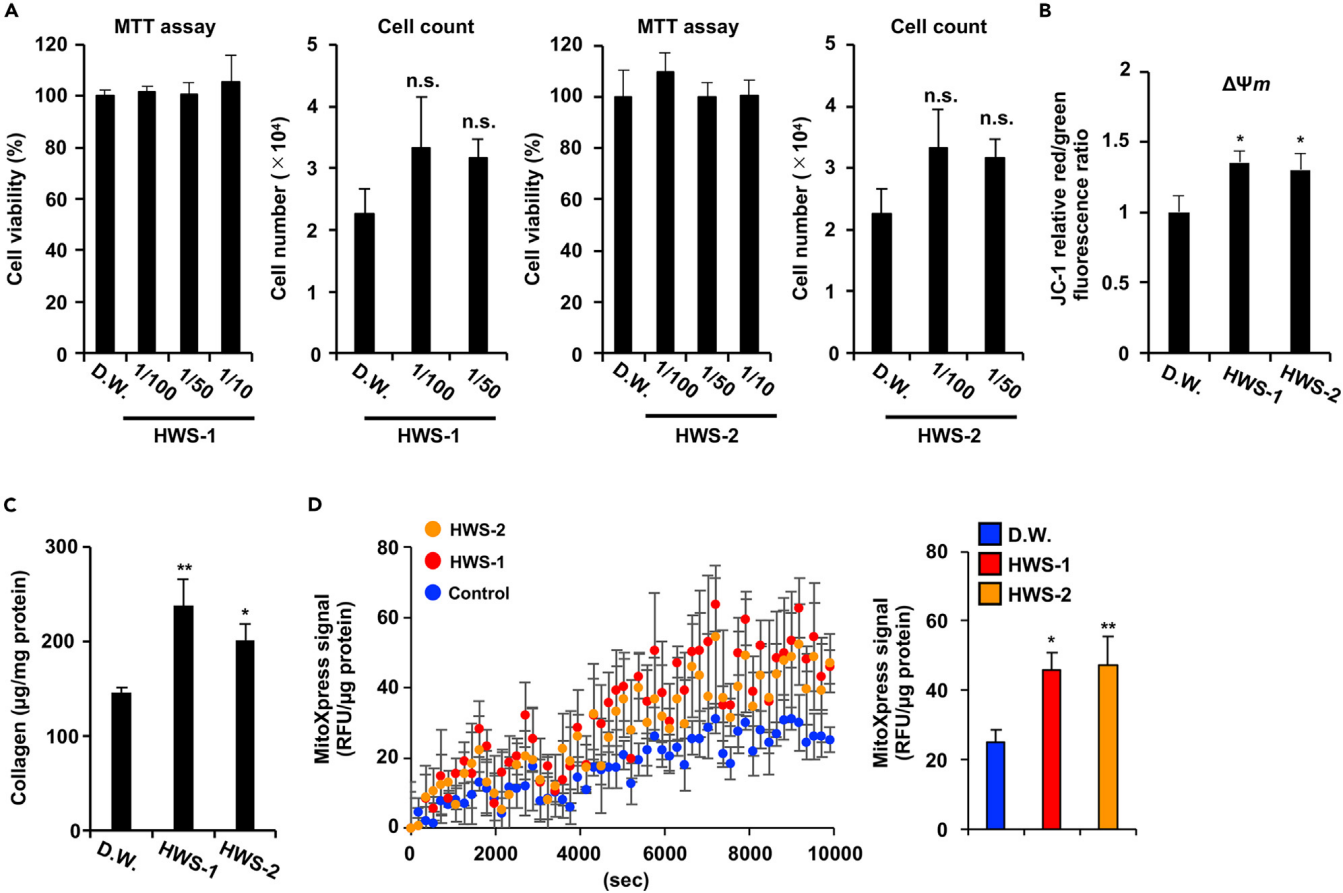
HWS stimulates collagen production (A) HWS did not exhibit cell toxicity. Cell viability was determined using the MTT assay and cell count assay. Distilled water (D.W.) was added to the control sample. (B, C) HWS induced collagen production and increased ΔΨ*m*. Aging NB1RGB cells were treated with or without HWS (1/100 diluted) for 48 h. (B) Microplate reader; ΔΨ*m* was determined by the ratio between the red fluorescence intensity, indicating activated mitochondria, and the green fluorescence intensity. (C) Collagen contents were measured. (D) Mitochondrial oxygen consumption is stimulated by HWS-1 or HWS-2 treatment. Aging NB1RGB cells were treated with HWS-1 or HWS-2 for 48 h. TR-F of the MitoXpress probe was performed using the same procedure described in Figure 1C (right panel), and the final measurement point was represented by a bar graph (right panel). Data are presented as the mean ± SD of three simultaneously performed experiments, three wells on the same plate (A: MTT assay, B, D), and different plate (A: Cell count assay, C). p values were calculated using ANOVA following Tukey-HSD test (A-D); n.s.: not significant, ∗p < 0.05, ∗∗p < 0.01.

Several reports have shown that mitochondrial dysfunction stimulates cellular aging,^3^^,^^20^ suggesting that the reactivation of mitochondrial function can suppress cellular aging. The hot water extract of *S. platensis* (HWS) has been reported to exert several biological effects on human cell lines.^13^ Thus, we herein aimed to determine whether HWS can reactivate mitochondrial function in aging fibroblasts. As shown in Figures 2A and 2B, two different culture lots of HWSs increased ΔΨ*m* in aging fibroblasts without inducing cell death. The results presented in Figure 1 indicate that collagen production decreased in a mitochondrial dysfunction-dependent manner. We examined whether HWS treatment increases collagen production, found that HWSs increased collagen production in aging NB1RGB (Figure 2C). To confirm whether HWS stimulates mitochondrial oxygen consumption, we treated aging NB1RGB and IMR90 cells with HWSs and analyzed mitochondrial oxygen consumption using an oxygen-sensitive fluorescent probe. As shown in Figures 2D and S4A, HWSs treatment increased mitochondrial oxygen consumption. These results indicate that HWS has the potential to improve mitochondrial function and therefore antiaging effects.

### SPC restores mitochondrial function and stimulates antioxidant activity

**Figure 3:**
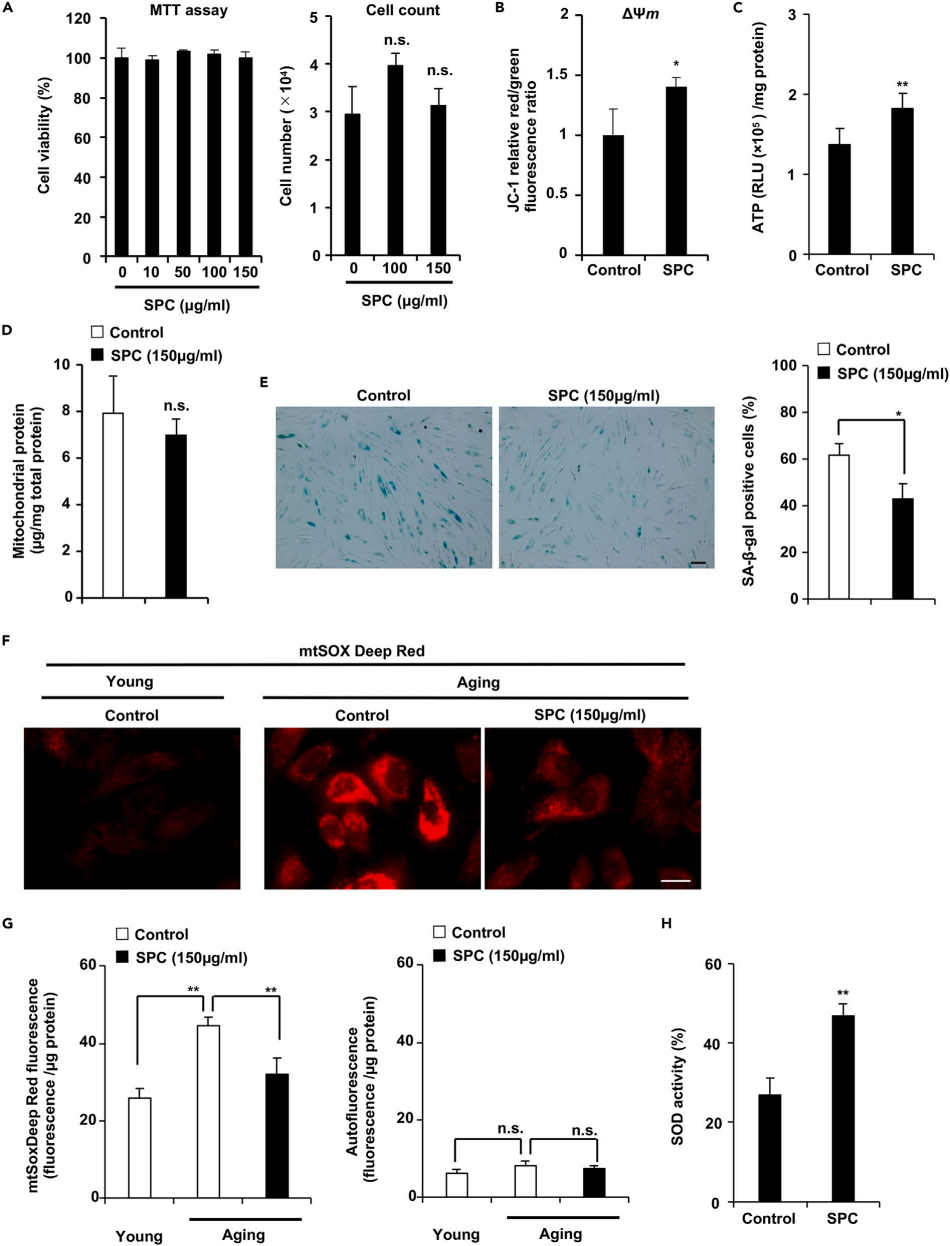
SPC restored mitochondrial activity and decreased ROS production (A) SPC did not exhibit cell toxicity. Cell viability was determined using the MTT assay and cell count assay. (B) SPC stimulated ΔΨ*m*. The cells were incubated with JC-1 for 20 min and subjected to a microplate reader. (C) SPC stimulated ATP production. ATP levels were determined using the CellTiter-Glo assay and normalized by amount of total protein. (D) The amount of mitochondrial protein did not affect by SPC treatment. Aging NB1RGB cells were treated with 150 μg/mL of SPC for 72 h and subjected to subcellar fractionation, then mitochondrial protein was determined by Bradford assay. (E) SPC decreased SA-β-gal positive cells. Aging NB1RGB cells were treated with 150 μg/mL of SPC for 72 h and subjected to SA-β-gal staining These images were taken at 10× magnification (scale bar, 50 μm) (right panel), and the 100–150 cells were counted, and the percentage of SA-β-gal positive cells among them was determined (n = 3) (left panel). (F–H) SPC eliminated ROS, which was increased in aging cells. Young and aging NB1RGB cells were treated with 150 μg/mL of SPC for 48 h. The ROS level in the cells was determined using the mtSOX Deep Red staining assay (F, G). (F) Fluorescence microscope image of staining of mtSOX Deep Red. These images were taken at 100× magnification (scale bar, 20 μm). (G) The fluorescence intensity of mtSOX Deep Red was determined after subtracting the autofluorescence intensity of the cells by using plate reader (left panel), and autofluorescence by using same measurement condition of mtSOX Deep Red were showed (right panel). (H) The anti-ROS activity was determined using an SOD assay kit. This assay is designed to evaluate the superoxide scavenging capacity of cells, which is generated by xanthine oxidase. Consequently, this method allows for the assessment of SOD activity. Data are presented as the mean ± SD of three simultaneously performed experiments, using three wells on the same plate (A-C, E-H) or different plate (D). p values were calculated using Student’s *t* test (B-E, H), ANOVA following Tukey-HSD test (A) and two-way ANOVA following Tukey-HSD test (G); n.s.: not significant, ∗p < 0.05, ∗∗p < 0.01.

The HWS contains SPC, which has several biological activities, such as immune system activation.^16^ However, whether SPC can activate mitochondrial function has not yet been elucidated. First, we verified the purity of the SPC used in our study. Our SPC contained 87 ± 1% carbohydrates as determined by a *Total organic carbon* (TOC) analyzer, or 77 ± 4% carbohydrates using the phenol-sulfuric acid method with a glucose standard, and 0.3 ± 0.15% protein. These data indicate that, although there is some discrepancy in the SPC measurement because of the methodology and calculations based on glucose, approximately 85% of the SPC is composed of carbohydrates and, with the remaining weight presumably consisting of water and other non-carbon components. Considering the high molecular weight of SPC (1,000–30,000), it is expected to exert its effects by interacting with receptors on the cell membrane rather than by entering the cell, as previously reported.^18^ As shown in Figures 3A, 3B, and 3C, SPC increased ΔΨ*m* and ATP production in aging fibroblasts without inducing cell death. Cellular aging and mitochondrial activation stimulate ROS production; thus, we aimed to determine whether SPC affects ROS cycling. We also investigated whether SPC influences mitochondrial content. Cells were subjected to a cell fractionation assay to isolate mitochondria. Our results demonstrated that SPC did not alter mitochondrial content (Figure 3D). Senescence associated-β-galactosidase (SA-β-gal) is important aging-related marker. In Figure 3E, the number of SA-β-gal-positive cells was decreased by treatment with SPC in aging NB1RGB cells, indicated that SPC suppresses the aging phenotype. As shown in Figure 3F, mtSOX Deep Red fluorescence signals increased in aging NB1RGB cells compared to young NB1RGB cells, and SPC treatment reduced these signals in aging NB1RGB cells. In Figure S1, autofluorescence signals were much weaker than mtSOX Deep Red signals in aging NB1RGB cells, indicating that autofluorescence signals did not affect the mtSOX Deep Red imaging results in Figure 3F. As demonstrated in Figure 3G (right panel), there was no significant difference in autofluorescence intensity between young and aging NB1RGB cells when using the emission and excitation filter combination for mtSOX Deep Red. The mtSOX Deep Red fluorescence intensity, after subtracting the cell autofluorescence value, increased in aging NB1RGB cells, and this increase was suppressed by SPC treatment (Figure 3G, left panel). We also confirmed mtSOX Deep Red fluorescence intensity was suppressed by SPC treatment in aging IMR90 cells (Figure S4B). Next, we measured the antioxidant activity, which is scavenging superoxide produced by xanthine oxidase, in SPC-treated aging NB1RGB cells and found that it was stimulated, indicated that SPC increased SOD protein expression and/or activity in aging NB1RGB cells (Figure 3H). These findings indicate that SPC can restore antioxidant activity and mitochondrial function in aging NB1RGB cells.

We examined the effect of SPC on young NB1RGB cells. As shown Figure S2A, SPC did not affect cell viability. The results of Figures S2B–S2D indicated that SPC did not affect collagen production, ROS generation, and SOD2 expression. These results suggested that possess sufficiently high levels of mitochondrial function and SOD2 expression that making them resistant to the effects of SPC treatment. Consequently, we focused our subsequent experiments only on aging cells.

### SOD2 is partially induced via the NF-κB pathway by SPC in aging fibroblasts

**Figure 4:**
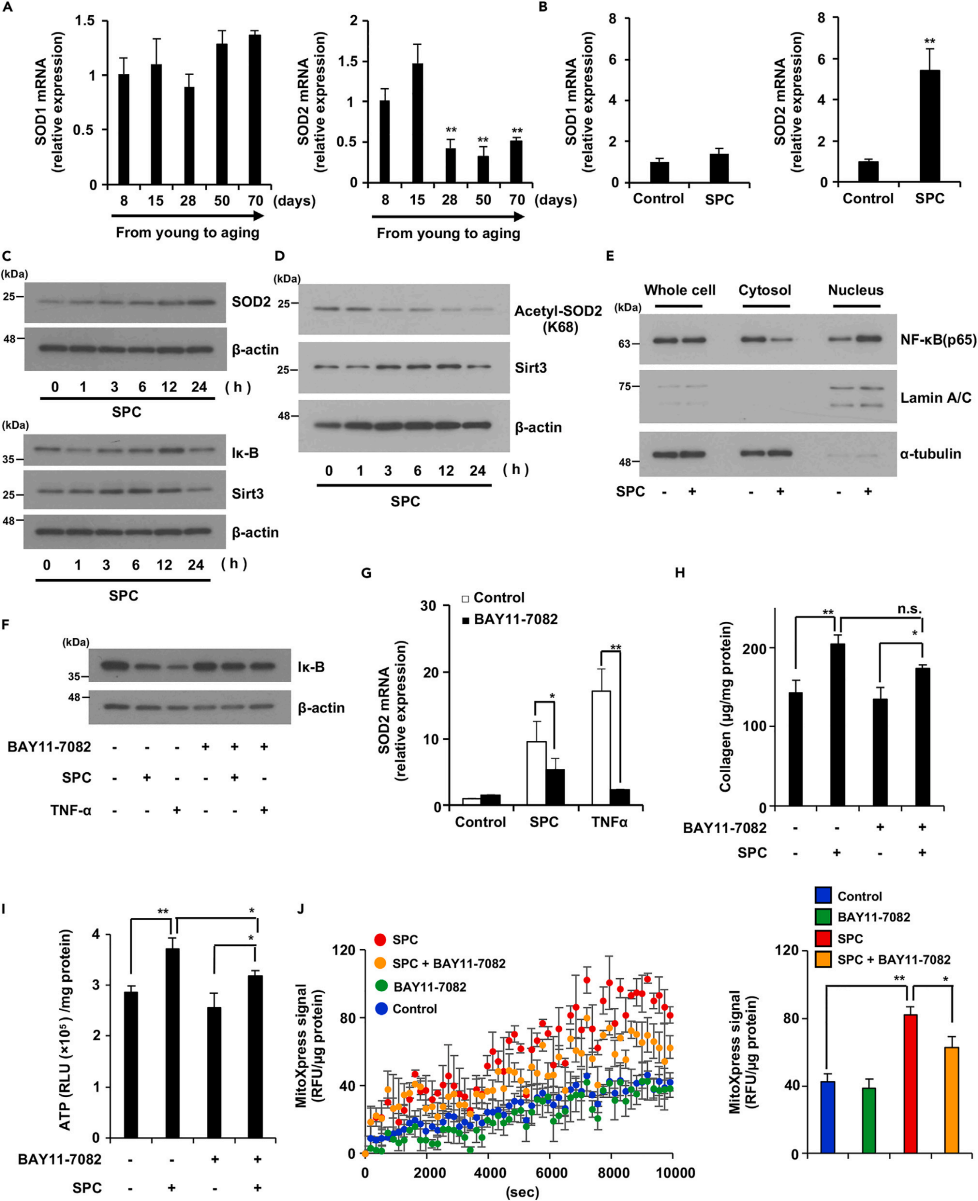
SPC upregulated SOD2 expression via the NF-κB pathway (A) SOD2 expression was decreased through the aging process. Total RNA was extracted from NB1RGB cells at several passages and subjected to real-time quantitative PCR. Cells were cultured for 8, 15, 28, 50, and 70 days. (B) SPC specifically upregulated SOD2 expression. Aging NB1RGB cells were treated with 150 μg/mL of SPC for 24 h; their RNA was then subjected to qPCR. (C and D) Aging NB1RGB cells were treated with 150 μg/mL of SPC for the indicated time periods. Cells were subjected to immunoblotting using the indicated antibodies. (C) SPC downregulated Iκ-B expression before upregulating SOD2. (D) SPC decreased acetyl-SOD2 expression via upregulation of Sirt3 expression. (E) SPC stimulated nucleus translocation of NF-κB (p65). Aging NB1RGB cells were treated with 150 μg/mL of SPC for 4 h and subjected to fractionation into three parts (whole cell, cytosol, and nucleus) and blotted with antibodies to NF-κB (p65), Lamin A/C (nucleus marker), and α-tubulin (cytosol marker). (F and G) Suppression of Iκ-B degradation partially inhibited the SPC-induced SOD2 upregulation. Aging NB1RGB cells were treated with or without 10 μM of BAY11-7082 and 150 μg/mL of SPC or 100 ng/mL of TNF-α for 1 h (left panel) or 24 h (right panel); they were then subjected to immunoblotting using the indicated antibodies (F) or qPCR (G). (H–J) Suppression of Iκ-B degradation partially inhibited the SPC-induced upregulation of ATP and oxygen consumption, but not collagen production. Aging NB1RGB cells were treated with or without 10 μM of BAY11-7082 and 150 μg/mL of SPC for 48 h (G, I) or 24 h (J); they were then subjected to collagen assay (H) ATP assay (I), and MitoXpress assay (J). Data are presented as the mean ± SD of three simultaneously performed experiments, using three wells on the same plate (I, J) or three wells on independent plates (A, B, G, H). p values were calculated using Student’s t test (B) and ANOVA following Tukey-HSD test (G-J); n.s.: not significant, ∗p < 0.05, ∗∗p < 0.01.

Several reports have indicated controversial results, i.e., SOD2 expression is higher in young fibroblasts than that in aging fibroblasts.^21^^,^^22^ Here, we found that the expression levels of SOD1 and SOD2 in NB1RGB cells changed in an age-dependent manner. As shown in Figure 4A, *SOD2* mRNA expression decreased in a time-dependent manner but not *SOD1* mRNA expression. Therefore, we hypothesized that SPC can recover *SOD2* expression levels in aging fibroblasts. To validate this hypothesis, we treated aging fibroblasts with and without SPC and then measured *SOD1* and *SOD2* mRNA expression levels in them. We found that SPC induced the expression of *SOD2* mRNA but not *SOD1* mRNA (Figure 4B). This finding suggested that SPC promotes the antioxidant capacity of cells by restoring the expression of *SOD2*, which is reduced by aging.

SOD2 activity is controlled by its expression level and acetylation.^23^^,^^24^ The SOD2 expression level is mainly controlled via the NF-κB pathway, wherein Iκ-B binds to NF-κB to inhibit its activation; furthermore, stimulants such as TNF-α and LPS can activate NF-κB by degrading Iκ-B.^24^ Acetyl-SOD2 is the inactivated form of SOD2; activation of SOD2 thus requires deacetylation of acetyl-SOD2 via Sirt3.^23^ As shown in Figures 4C, 4D, and S4C, aging NB1RGB and IMR90 cells treated with SPC had increased SOD2 and Sirt3 expression, and decreased Acetyl-SOD2 expression; however, Iκ-B expression was decreased in aging NB1RGB cells before the increase in SOD2 expression. To verify whether the NF-κB pathway is activated by SPC treatment, we examined the cellular translocation of NF-κB (p65) into the nucleus in response to SPC treatment using cell fractionation methods. As demonstrated in Figures 4E and S5B, SPC treatment stimulated the subcellular localization of NF-κB (p65) from the cytosol to the nucleus. These results indicate that the SPC-induced increase in SOD2 expression is related to NF-κB signaling, and the deacetylation of acetyl-SOD2 by Sirt3. We also confirmed that HWSs induced SOD2 expression in aging NB1RGB and IMR90 cells (Figure S3). Furthermore, we examined whether the activation of NF-κB signaling was regulated via SPC-dependent SOD2 upregulation using BAY11-7082 ((2E)-3-[(4-Methylphenyl)sulfonyl]acrylonitrile), an inhibitor of Iκ-B degradation. Aging fibroblasts were first treated with or without BAY11-7082, followed by treatment with SPC or TNF-α. SPC and TNF-α decreased Iκ-B expression; however, BAY11-7082 suppressed SPC- and TNF-α-dependent Iκ-B degradation (Figure 4F). Moreover, SPC-induced SOD2 upregulation was partially suppressed by BAY11-7082, whereas TNF-α-induced SOD2 upregulation was almost completely suppressed by BAY11-7082 (Figure 4G). Next, we investigated the effect of an Iκ-B degradation inhibitor on collagen production, ATP, and oxygen consumption. SPC-induced upregulation of ATP and oxygen consumption was partially suppressed by BAY11-7082, but collagen production remained unaffected in aging NB1RGB cells (Figures 4H–4J). In aging IMR90 cells, BAY11-7082 treatment only suppressed SPC-induced ATP production, whereas collagen and oxygen consumption displayed a trend toward inhibition, although the difference was not significant (Figures S6A–S6C). These results suggest that SPC-induced upregulation of SOD2 expression is partially regulated by NF-κB signaling, and that NF-κB signaling also plays a partial role in restoring the reduced function of aging cells. However, pathways other than NF-kB signaling might also be involved in SPC-dependent SOD2 induction and antiaging effects.

### SPC does not elicit a potent inflammatory response similar to that induced by LPS

**Figure 5:**
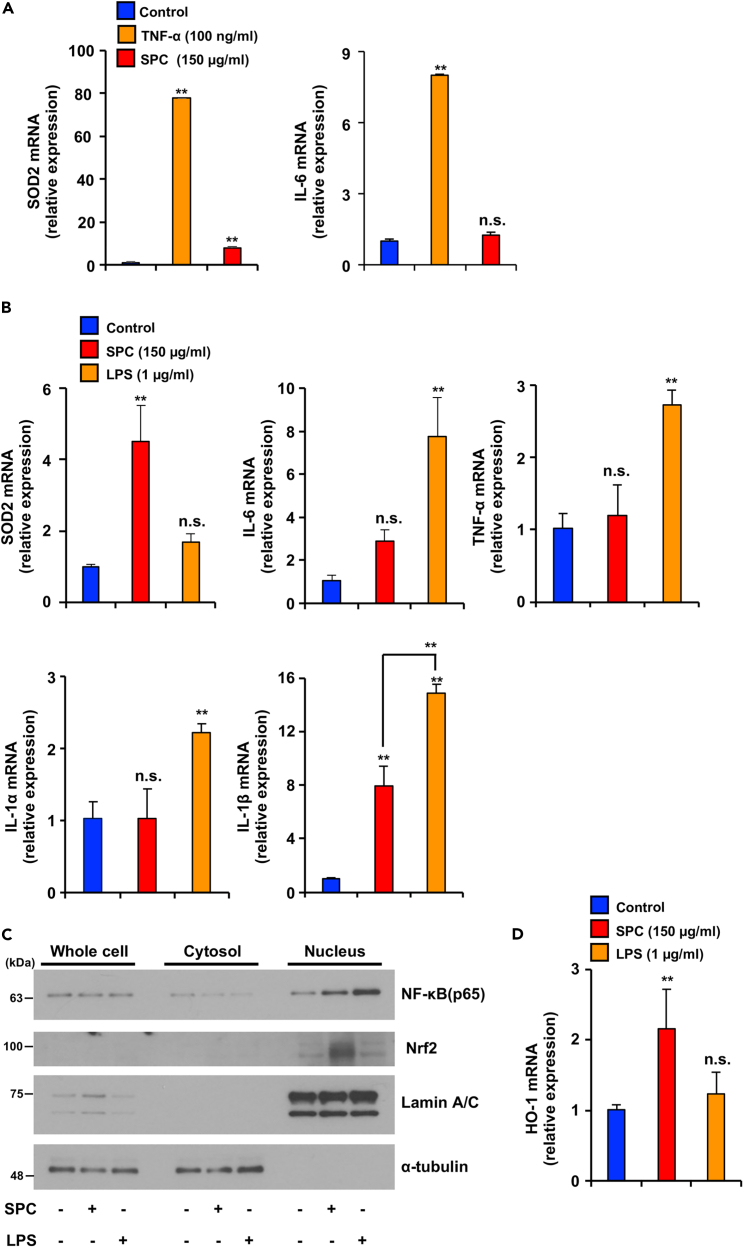
SPC did not activate inflammatory signaling (A–D) Aging NB1RGB cells were treated with SPC, TNF-α, or LPS at the indicated concentrations for 24 h (A, B, D) or 4 h (C); they were then subjected to qPCR (A, B, D) or fractionation assay using the same procedure described in Figure 1C. Data are presented as the mean ± SD of three simultaneously performed experiments, using three wells on independent plates (A-D). p values were calculated using ANOVA following Tukey-HSD test (A-D); n.s.: not significant, ∗p < 0.05, ∗∗p < 0.01.

Several reports have shown SOD2 upregulation by stimuli that induce inflammatory signals, such as LPS and TNF-α.^25^^,^^26^ Because SPC also induced SOD2, we aimed to determine whether SPC activates inflammatory signals by measuring the expression level of IL-6, TNF-α, IL-1α and IL-1β, an inflammatory related cytokine induced by inflammatory signals, in aging fibroblasts. As shown in Figure 5A, TNF-α upregulated SOD2 and *IL-6* mRNA in aging NB1RGB cells. SPC treatment did not induced IL-6, TNF-α, and IL-1α mRNA expression, however LPS treatment increased those of mRNA expression in aging cells (IL-6 and TNF-α mRNA expression did not detected our experiment in aging IMR90) (Figures 5B and S5A). The IL-1β mRNA expression was increased by SPC treatment but its expression level was significantly low compared to LPS treatment in aging NB1RGB and IMR90 cells (Figures 5B and S5A). These results suggest that LPS induces inflammatory signaling and upregulation of SOD2 expression in aging fibroblasts; however, SPC upregulates SOD2 expression without inducing most inflammation-related cytokines.

Previously reported that NF-E2-related factor 2 (Nrf2) is one of the important SOD2 inducible transcriptional factor, which is maintained at low expression levels in the cytoplasm because of degradation, and suppresses some inflammation-related cytokines such as IL-6 and TNF-α.^27^^,^^28^ Therefore, we investigated whether there is a difference in Nrf2 and NF-κB activation between SPC and LPS treatment. As shown in Figures 5C and S5B, Nrf2 was translocated in nucleus by treatment of SPC but not treatment of LPS in aging cells. NF-κB (p65) was translocated in nucleus with SPC and LPS treatment in aging cells. HO-1 is major Nrf2 target gene.^28^ SPC treatment upregulated HO-1 mRNA expression but not LPS treatment in aging cells (Figures 5D and S5C). Regarding the SPC-induced SOD2 induction and mild inflammatory pathway activation, it was suggested that SPC activates both NF-κB and Nrf2, leading to a robust induction of SOD2, whereas the inflammatory pathway might be suppressed by Nrf2.

### SPC activates ER protein folding activity

**Figure 6:**
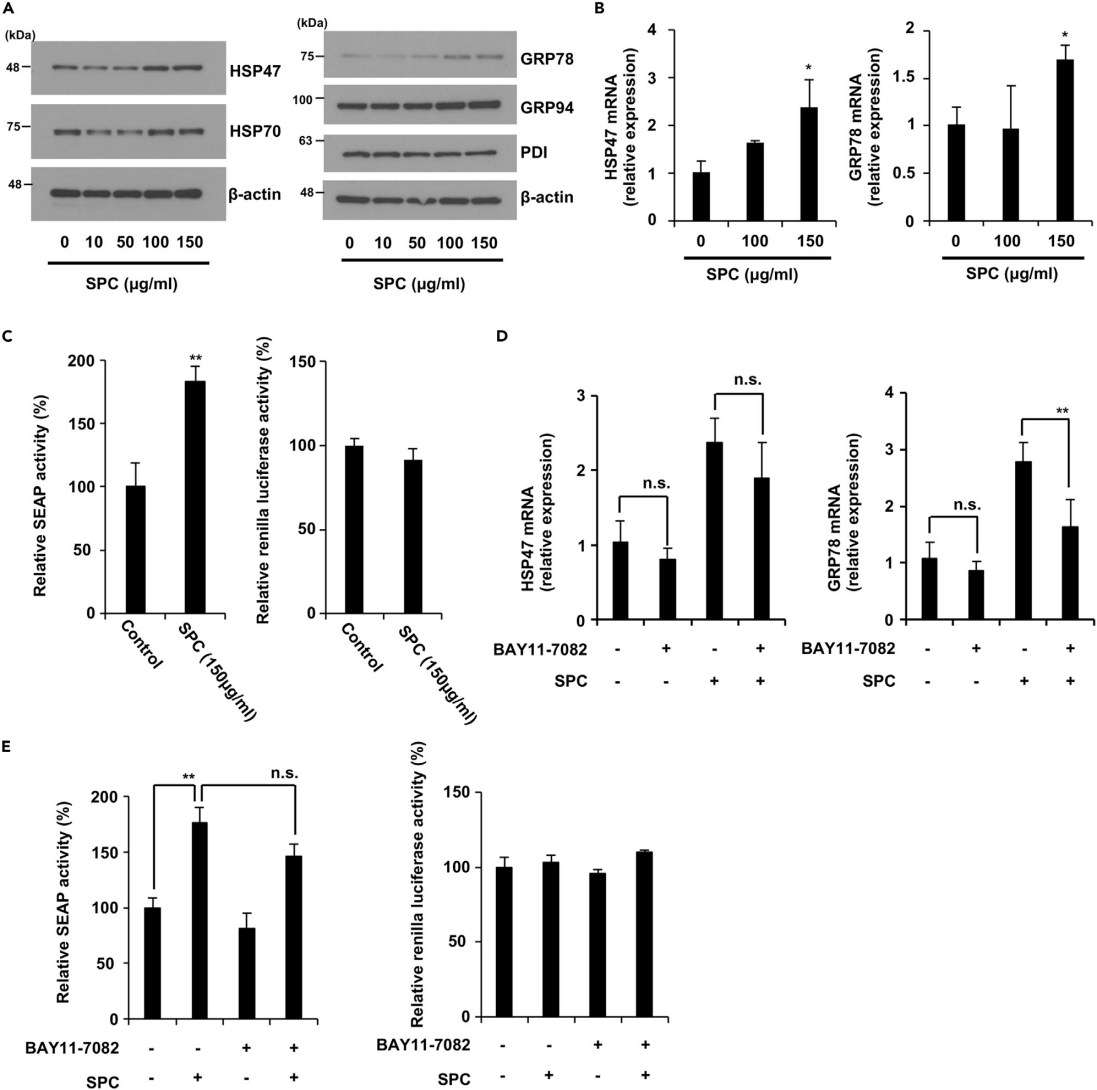
SPC stimulated ER secretory function (A and B) SPC upregulated HSP47 and GRP78 expression. Aging NB1RGB cells were treated with the indicated concentration of SPC for 24 h and then subjected to immunoblotting using the indicated antibodies (A) or qPCR (B). (C) Aging NB1RGB cells expressing SEAP were transduced with a pSEAP2-Control vector and washed 18 h after transduction; the cells were then treated with or without 150 μg/mL of SPC for 24 h. The medium was then changed, and the cells were cultured for another 12 h. Culture media were then analyzed for SEAP activity, and luminescence was normalized to cell number (left panel). Aging NB1RGB cells expressing Renilla luciferase were transduced with a pRL-Renilla luciferase control vector and washed 18 h after transduction; the cells were then treated with or without 150 μg/mL of SPC for 24 h (right panel). The data as percentages represented the RLU of each sample divided by the RLU of non-treated cells sample. (D and E) Suppression of Iκ-B degradation partially inhibited the SPC-induced upregulation of GRP78 expression, but not HSP47 expression and SEAP activity. Aging NB1RGB cells were treated with or without 150 μg/mL of SPC and/or 10 μM of BAY11-7082 for 24 h and then subjected to qPCR (D). (E) Aging NB1RGB cells expressing SEAP or Renilla luciferase were transduced with a pSEAP2-Control vector or pRL-Renilla luciferase control vector and washed 18 h after transduction; the cells were then treated with or without 150 μg/mL of SPC and/or 10 μM of BAY11-7082 for 24 h and same procedure in Figure 6C. Data are presented as the mean ± SD of three simultaneously performed experiments, using three wells on the same plate (C, E) or different plate (B, D). p values were calculated using Student’s *t* test (C) and ANOVA following Tukey-HSD test (B, D, E); n.s.: not significant, ∗p < 0.05, ∗∗p < 0.01.

ER function is an important factor involved in the regulation of collagen production; proper collagen construction depends on ER chaperones, such as heat shock protein 47 (HSP47) and glucose-regulated protein 78 (GRP78). Our results suggested that SPC increases collagen production by upregulating SOD2 expression; thus, we examined how SPC affects ER function. SPC treatment increased the HSP47 and GRP78 mRNA and protein expression of HSP47 and GRP78 in aging fibroblasts but did not affect the protein expression levels of HSP70 (cytosol chaperone), GRP94, and PDI (ER chaperone), suggesting that SPC may increase ER protein folding capacity (Figures 6A and 6B). Therefore, we investigated the effect of SPC on the secretory output of the ER using the secreted alkaline phosphatase (SEAP) assay.^29^ We expressed SEAP in aging fibroblasts and monitored its secretion. SPC significantly stimulated the secretion of SEAP (Figure 6C left panel). To exclude the potential impact of SPC on transcription efficiency of SEAP gene, we measured pRL-Renilla Luciferase activity, SPC did not affect Renilla Luciferase activity in aging NB1RGB cells (Figure 6C, right panel). Consequently, the enhancement of collagen production by SPC might be related to the stimulation of the ER secretory function.

Next, we investigated the effect of an Iκ-B degradation inhibitor on HSP47 and GRP78 mRNA expression, and SEAP activity. SPC-induced upregulation of GRP78 mRNA was partially suppressed by BAY11-7082, but HSP47 mRNA remained unaffected in aging NB1RGB cells (Figure 6D). The SEAP activity exhibited a trend toward suppression, but not significantly in aging NB1RGB and IMR90 cells (Figures 6E and S6D). These findings suggest that NF-κB signaling is unlikely to directly enhance the ER folding capacity.

### SPC stimulates collagen production via the upregulation of SOD2 in aging fibroblasts

**Figure 7:**
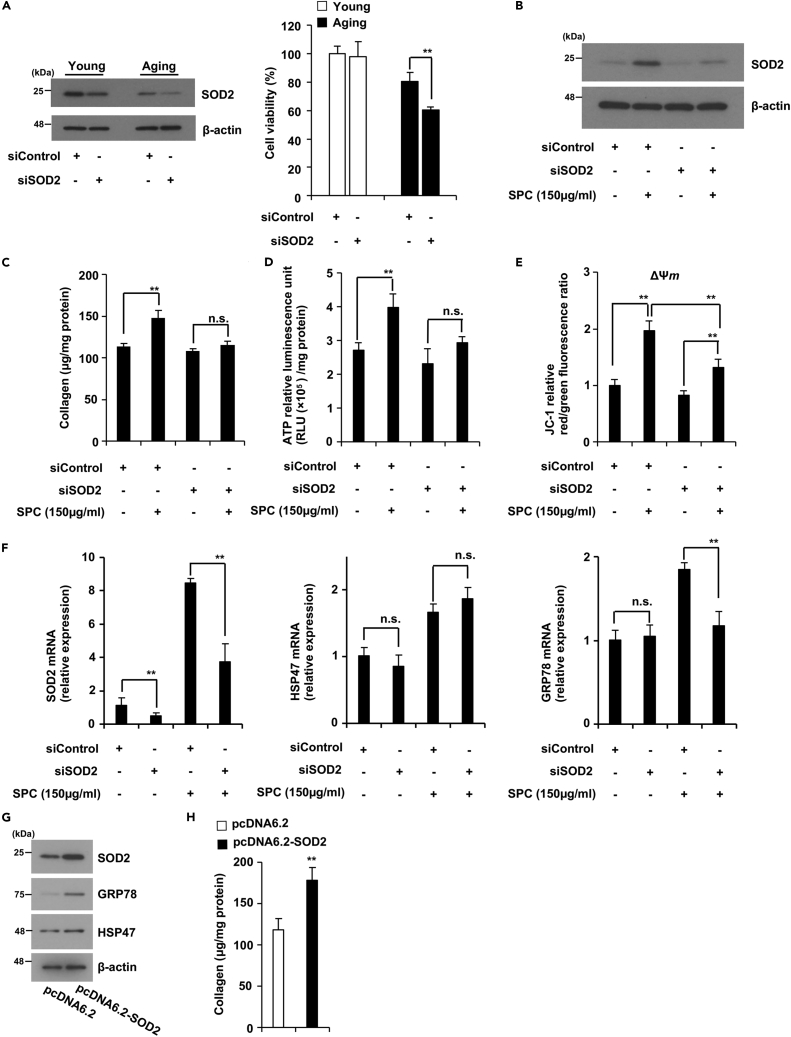
SPC-induced increase in collagen production was dependent on the upregulation of SOD2 expression (A) The SOD2 expression level played an important role in cell viability in aging NB1RGB cells. Aging or young NB1RGB cells were transfected with 25 nM of siControl or siSOD for 24 h. Cells were subjected to immunoblotting using the indicated antibodies (left panel) or MTT assay (right panel). (B–E) SPC-dependent upregulated collagen production, ΔΨ*m* and ATP production were suppressed by the downregulation of SOD2 expression. Aging NB1RGB cells were transfected with 25 nM of siControl or siSOD2 for 24 h, followed by treatment with or without 150 μg/mL SPC for 48 h. Cells were subjected to immunoblotting using the indicated antibodies (B), measurement of collagen content (C), ΔΨ*m* (D) or ATP production (E). (F) SPC-dependent upregulated GRP78 expression was suppressed by the downregulation of SOD2 expression but not HSP47 expression. Aging NB1RGB cells were transfected with 25 nM of siControl or siSOD2 for 24 h, followed by treatment with or without 150 μg/mL SPC for 24 h. Cells were subjected to qPCR. (G and H) Over-expression of SOD2 stimulated GRP78 expression and collagen production in aging NB1RGB cells. Aging NB1RGB cells were transfected with pcDNA6.2 empty vector or pcDNA6.2-SOD2 for 48 h. Cells were subjected to immunoblotting using the indicated antibodies (G), measurement of collagen content (H). Data are presented as the mean ± SD of three simultaneously performed experiments, using three wells on the same plate (A, D, E) or different plate (C, F, H). p values were calculated using Student’s *t* test (H) and two-way ANOVA following Tukey-HSD test (A, C-F); n.s.: not significant, ∗p < 0.05, ∗∗p < 0.01.

Next, we aimed to determine whether SPC restores collagen production in aging fibroblasts by upregulating SOD2 expression. Young and aging fibroblasts were first treated with SOD2 siRNA (siSOD2) for 24 h. Then, the treated cells were subjected to immunoblotting and MTT assay. As shown in Figure 7A, siSOD2 suppressed the SOD2 basal expression level in both young and aging fibroblasts, and SOD2 expression was lower in aging fibroblasts than that in young fibroblasts. SOD2 knockdown decreased cell viability in aging fibroblasts, indicating that the survival of aging fibroblasts depends on the expression level of SOD2. We investigated whether SPC-induced increases in collagen production and mitochondrial function are dependent on SOD2 induction. As shown in Figure 7B, siSOD2-treated aging NB1RGB cells exhibited suppressed SPC-induced SOD2 expression. SPC stimulated collagen and ATP production in siControl treated cells, but did not affect collagen and ATP production in siSOD2 treated cells (Figures 7C and 7D). In aging IMR90 cells, SPC also did not influence ATP production in siSOD2 treated cells (Figure S7B). The ΔΨ*m* was increased by SPC in both siControl and siSOD2-treated cells; however, siSOD2 significantly suppressed ΔΨm upregulation by SPC (Figure 7E). These results indicate that SPC-dependent upregulation of SOD2 plays a crucial role in SPC-stimulated collagen production and mitochondrial function.

We demonstrated the effects of HSP47 and GRP78 expression by knockdown of SOD2, and the impact of HSP47 and GRP78 expression and collagen production by overexpression of SOD2. As shown in Figures 7F and S7A, knockdown of SOD2 suppressed SPC-induced GRP78 mRNA expression but not HSP47 mRNA expression in aging NB1RGB and IMR90 cells. Overexpression of SOD2 stimulated GRP78 expression and collagen production, but HSP47 expression remained unchanged in aging NB1RGB cells (Figures 7G and 7H). These results suggest that collagen production and GRP78 expression are related to SOD2 expression, whereas HSP47 expression levels are not controlled by SOD2 expression levels.

## Discussion

In this study, we found that HWS stimulated mitochondrial function and collagen production. Moreover, SPC was identified as the component of this extract that potentially conferred these activities. SPC restored mitochondrial function by scavenging ROS and inducing collagen production via the upregulation of SOD2, the expression of which is downregulated in an age-dependent manner in aging fibroblasts. Analysis of the molecular mechanism underlying SPC activity indicated that it induces SOD2 by decreasing Iκ-B expression and thereby activating NF-κB signaling. We also found that SPC induced SOD2 but not the inflammatory cytokine IL-6, which was induced by LPS and TNF-α, indicating that SPC has strong antioxidant activity but does not induce inflammatory signals. Moreover, the treatment of fibroblasts with SPC stimulated ER secretory activity via the upregulation of several ER chaperones. Thus, SPC plays a central role in the antiaging effect mediated by SOD2 induction in aging fibroblasts via NF-κB signaling and not inflammatory signaling.

Many reports have suggested that SOD2 is important for scavenging the superoxide produced in mitochondria, with impaired SOD2 function causing mitochondrial dysfunction, which leads to aging-related neurodegenerative diseases and liver disorders. Studies have also indicated that heterozygote deficiency of SOD2 promotes whole-body aging in mice.^5^^,^^6^^,^^30^ Thus, SOD2 dysfunction is related to the aging process. Sirt3 is an important antiaging target, and one of its functions is to activate SOD2 via deacetylation of acetyl-SOD2.^31^ Although analysis of the expression and function of SOD2 in the aging process is limited, we found that the expression and activity of SOD2 decreased in a replication- and aging-dependent manner in aging fibroblasts, inducing mitochondrial dysfunction related to the cellular aging phenotype. These findings demonstrate that SOD2 is a potential antiaging target. The expression of Sirt3 and SOD2 proteins occurs at different time points following SPC treatment, as shown in Figures 4C and 4D. Our results suggest that SPC-induced SOD2 protein expression is partially dependent on the NF-kB pathway. However, the upregulation of Sirt3 protein may not be primarily related to the NF-kB pathway, and an SPC-induced, yet undetermined, signaling pathway could be involved.

We predict that SPC, which is a high molecular weight compound, activates the NF-κB pathway via a receptor in the cellular membrane to increase the SOD2 protein expression level in aging cells. TNF-α and LPS, which are well-known SOD2 inducers, increased SOD2 expression in aging fibroblasts in the present study; however, differences between SOD2 induction by SPC and that by TNF-α and LPS were noted. SPC might selectively induce SOD2 rather than by activating inflammatory pathways, with SPC-dependent activation of Nrf2 potentially playing a significant role in these phenomena; however, further studies are needed to confirm this hypothesis and understand the underlying processes in more detail.

An important factor in collagen production is the formation of specific structures in the ER. HSP47 specifically binds to and stabilizes collagen, and the proper function of HSP47 is essential for the correct folding and assembly of collagen molecules. Although GRP78 is not specific to collagen, it assists in the folding of various proteins, including collagen.^9^ Consequently, a decrease in ER chaperone-dependent protein folding activity may lead to suppressed collagen production.^32^ In addition, it has also been reported that decreased mitochondrial function suppresses collagen production and stimulates collagen degradation.^33^ Here, we found that SPC stimulated collagen production by activating mitochondrial function, and upregulating the expression of ER chaperones and enhancing the ER protein folding capacity.

*S. platensis* fixes atmospheric carbon through photosynthesis to produce various carbon-based compounds and contains abundant amounts of various proteins^13^; thus, several studies have focused on exploring *S. platensis* as an environmentally sustainable source of nutrients. In this study, we focused on an application of SPC, which can be separated from other *S. platensis* nutrients. SPC has been reported to modulate immunological activity and exhibit antitumor activity.^15^ Here, we investigated the antiaging effect of SPC via mitochondrial activation in aging fibroblasts. Owing to ongoing research on the value or applicability of SPC, the use and production of *Spirulina* is expected to increase and address environmental issues to a certain extent.

Overall, our findings revealed that SOD2 expression decreases in the aging process, which is induced by mitochondrial dysfunction. SPC restores mitochondrial function via the upregulation of SOD2 expression. Thus, SPC can serve as a promising antiaging material.

### Limitations of the study

In the present study, we elucidated the anti-aging effects of SPC on aging fibroblasts. We used proliferative and replicative aged NB1RGB and IMR90 cells for our experiments. Therefore, analyses involving other aging models or other cell types are warranted to further extend the results of this study. Although SPC is certainly weaker than the LPS-induced inflammatory signal, it has not been tested whether it suppresses all inflammatory cytokines. Finally, the exact mechanisms by which SPC triggers activation of the NF-κB and Nrf2 pathways and induction of GRP78 and HSP47 remain elusive, particularly the specific factors directly targeted by SPC. Further research is required to fully understand these mechanisms.

## STAR★Methods

### Key resources table

**Table:** 

REAGENT or RESOURCE	SOURCE	IDENTIFIER
**Antibodies**		

GRP78	Cell Signaling	Cat # 3177; RRID: AB_2119845
GRP94	Cell Signaling	Cat # 2104; RRID: AB_823506
HSP70	Cell Signaling	Cat # 4872; RRID: AB_227984
Iκ-B	Cell Signaling	Cat # 4814; RRID: AB_390781
PDI	Cell Signaling	Cat # 3501; RRID: AB_2156433
Sirt3	Cell Signaling	Cat # 2627; RRID: AB_2188622
Lamin A/C	Cell Signaling	Cat # 4777; RRID: AB_10545756
Nrf2	Cell Signaling	Cat # 12721; RRID: AB_2715528
NF-κB(p65)	Cell Signaling	Cat # 8242; RRID: AB_10859369
SOD2	Cell Signaling	Cat # 13141; RRID: AB_2636921
HSP47	Abcam	Cat # ab109117; RRID: AB_10888995
Acetyl-SOD2(K68)	Abcam	Cat # ab137037; RRID: AB_2784527
β-actin	sigma	Cat # A5441; RRID: AB_476744
α-tubulin	sigma	Cat # T9026; RRID: AB_477593
anti-rabbit-HRP	Promega	Cat # W4011; RRID: AB_430833
anti-mouse-HRP	Promega	Cat # W4021; RRID: AB_430834

**Chemicals, peptides, and recombinant proteins**		

MEMα	Wako	Cat # 135-15175
JC-1	Dojindo	Cat # 349-09401
MitoXpress oxygen-sensitive probe	Abcam	Cat # ab197243
MTT	Dojindo	Cat # M009
BAY11-7082	Wako	Cat # 020-17871
Trypan Blue solution	Sigma	Cat # T8154
mtSOX Deep Red	Dojindo	Cat # MT14
MitoSOX Red	Invitrogen	Ca t# M36008
THUNDERBIRD® SYBR® qPCR Mix	TOKOBO	Cat # QPS-201
Lipofectamine 3000	Invitrogen	Cat # L3000001
RNAiMAX	Invitrogen	Cat # 13778150

**Critical commercial assays**		

Total Carbohydrate Assay kit	CELL BIOLABS	Cat # #STA-682
senescence detection kit	Abcam	Cat # ab65351
collagen quantitation kit	Cosmo Bio	Cat # COL-001
CellTiter-Glo 2.0 assay kit	Promega	Cat # G9241
SOD Assay Kit-WST	Dojindo	Cat # S311
Great EscAPe SEAP Reporter System	Takara Bio	Ca t# 631736
Dual-Luciferase Assay system	Promega	Cat # E1910

**Experimental models: Cell lines**		

NB1RGB	Riken BRC	Cat # RCB0222
IMR90	JCRB Cell Bank	Cat # JCRB9054

**Recombinant DNA**		

pENTR221-SOD2	DNAFORM	Cat # 100008212

**Software and algorithms**		

Mac statistical analysis software	Esumi	Cat # Mac statistical analysis Ver. 3

### Resource availability

#### Lead contact

Further information and requests for resources and reagents should be directed to and will be fulfilled by the lead contact, Takushi Namba (t-namba@kochi-u.ac.jp).

#### Materials availability

Resources and materials will be provided upon reasonable request, considering the terms of the materials transfer agreement (MTA) for the modified reagents. We may require a completed MTA if there is potential for commercial application.

### Experimental model and subject details

#### Cell lines

NB1RGB (human skin fibroblast) (Riken BRC) and IMR90 (human lung fibroblast) (JCRB Cell Bank) cells were maintained in MEMα (Wako, Tokyo, Japna) supplemented with 10% FBS, 100 U/ml penicillin, and 100 μg/ml streptomycin. Cell cultures were passaged (1:4, every 3 days) and maintained at 37°C under 5% CO_2_. We defined two types of each cell line according to the number of days in culture: NB1RGB: young cells: from 8 to 20 days and aging cells: from 60 to 70 days; IMR90: young cells: from 6 to 12 days and aging cells: from 30 to 40 days^34^

NB1RGB cells were transiently transfected using lipofection (Lipofectamine 3000, Invitrogen, Carlsbad, CA, USA) with plasmids expressing SOD2 (pcDNA6.2-SOD2, cloned from pENTR221-SOD2 (DNAFORM ID 100008212) using Gateway cloning system (Invitrogen, Carlsbad, CA, USA).)

### Method details

#### The hot water extract of spirulina platensis

Spray-dried powder of *S. platensis* (DIC Co., Ltd. Tokyo, Japan) cultivated under basic conditions (pH 11) in outdoor open ponds was extracted with water in an autoclave for 1 h at 120°C. The water-soluble extract was prepared by removal of insoluble fractions by centrifugation.^35^ We used two different culture lots, which are HWS-1 and HWS-2. HWS-1: water-95.8%, solid content-4.2%. HWS-2: water-95.7%, solid content-4.3%.

#### The preparation of SPC

SPC fraction was prepared from *S. platensis* dried cells according to the method described.^18^ Briefly, cells were washed by acetone, suspended in distilled water (D.W.), and then extracted by an addition of 90% phenol-water at 68°C. The crude preparation was dialyzed to remove phenol and then freeze-dried. The sample was dissolved in the water and precipitate was eliminated by centrifugation. The molecular mass of the sample was in between 1000 and 20,000 by electrophoresis. SPC was dissolved in distilled water (D.W.).

#### Total Carbohydrate Assay

Total carbohydrates were quantified using the Phenol Sulfuric Acid method (Total Carbohydrate Assay kit #STA-682 (CELL BIOLABS, INC. San Diego, CA, USA)) and the experiment was performed according to the manufacturer’s protocol. SPC and standards were mixed with 5% phenol solution, followed by the addition of concentrated sulfuric acid. After incubation, absorbance was measured at 490 nm. Carbohydrate content was calculated using a calibration curve derived from glucose standards.

#### *Total organic carbon (*TOC) analysis

The carbon concentration of SPC was measured using a TOC-VCPN analyzer (Shimadzu Corp., Kyoto, Japan) after dissolving the SPC (4 mg) in water (20 mL) and Carbohydrate amounts were calculated using glucose molecular weight.^36^

#### Detection of SA-β-gal activity

Cellular SA-β-Gal activity was assessed using a senescence detection kit #ab65351 (Abcam, Cambridge, England) according to the manufacturer’s protocols. All images were obtained under a microscope (IX73, Olympus, Tokyo, Japan) and processed using the Adobe Photoshop software.

#### JC-1 staining

Mitochondrial membrane potential (ΔΨ*m*) was determined using JC-1 (Dojindo, Tokyo, Japan) staining according to the manufacturer’s protocols and a previous report.^37^ The results for JC-1 are shown as the ratio of fluorescence measured at 535 nm/590 nm to that measured at 485 nm/535 nm (aggregate fluorescence to monomer fluorescence) using a fluorescence microplate reader and i-control 1.11 software (Infinite M200, TECAN, Tokyo, Japan). All images were obtained under a fluorescence microscope (BZ-X800, KEYENCE, Osaka, Japan) and processed using the Adobe Photoshop software.

#### MitoXpress assay to measure oxygen consumption

Oxygen consumption was monitored using the MitoXpress oxygen-sensitive probe (Abcam, *Cambridge*, England) according to the manufacturer’s protocols and previous reports.^19^ Briefly, 1 μM of MitoXpress was added to each well, wells were sealed with 100 μl of mineral oil, and fluorescence was quantified at 37°C; time-resolved fluorescence measurements were performed at 380-nm emission and 650-nm excitation with a delay of 30 μs and gate time of 100 μs using a fluorescence microplate reader and i-control 1.11 software (Infinite M200, TECAN, Tokyo, Japan).

#### Collagen assay

Collagen levels were assayed using a collagen quantitation kit #COL-001 (Cosmo Bio, Tokyo, Japan) following the manufacturer’s instructions. Collagen production was normalized by total protein content. Fluorescence was measured using a fluorescence microplate reader (Infinite M200, Tecan, Tokyo, Japan) with a 360/485-nm filter pair.

#### ATP assay

Intracellular ATP levels were measured using the CellTiter-Glo 2.0 assay kit #G9241 (Promega, Madison, WI, USA) following the manufacturer’s instructions. ATP production was normalized by total protein content.

#### Cell viability assay

Cell viability was determined using the MTT (3-(4,5-Dimethylthiazol-2-yl)-2,5-Diphenyltetrazolium Bromide) assay and Cell count assay. In brief, MTT assay: cells were administered the indicated treatments and incubated with MTT solution (1 mg/mL) for 2 h. Isopropanol/HCl was then added to a final concentration of 50%/20 mM, and absorbance at 570 nm was measured using a spectrophotometer. Cell count assay: cells were harvested, resuspended in fresh culture medium, and stained with a 0.4% Trypan Blue solution (1:1 dilution) (Sigma, St. Louis, MO, USA). The stained cell suspension was loaded onto a hemocytometer and examined under a light microscope. Viable and non-viable cells were counted, and cell viability was calculated as the percentage of viable cells to the total number of cells.

#### Subcellar fractionation

Cells were resuspended in fractionation buffer and stored on ice for 30 min and then disrupted via passage through 26-gauge needles 15 times. Cell lysates were centrifuged at 720*g* for 5 min to isolate the nuclei fraction. The supernatant was centrifuged at 15,000*g* for 5 min. The pellet was retained as the mitochondrial fraction. Supernatant was centrifuged at 15,000*g* for 5 min, and subsequently, supernatants were collected as the cytosol fraction.

#### ROS measurement

Cells were first cultured in black-bottom or clear-bottom culture plates. They were then administered the indicated treatments and washed with phosphate-buffered saline (PBS) to remove the medium. The cells were subsequently incubated for 10 min at 37°C in 10 μM mtSOX Deep Red (Dojindo, Kumamoto, Japan) or MitoSOX Red (Invitrogen, Carlsbad, CA, USA).^38^ After incubation, the cells were again washed with PBS. Fluorescence was measured using a microplate reader with a 535/670-nm or 535 nm/590 nm filter pair using a fluorescence microplate reader and i-control 1.11 software (Infinite M200, TECAN, Tokyo, Japan). Determine the corrected fluorescence intensity by subtracting the cell autofluorescence value from the fluorescence intensity of cells treated with mtSOX Deep Red. All images were obtained under a fluorescence microscope (BZ-X800, KEYENCE, Osaka, Japan).

#### SOD assay

SOD levels were detected using the SOD Assay Kit-WST #S311 (Dojindo, Kumamoto, Japan) following the manufacturer’s instructions. The SOD activity of each sample was standardized using intracellular protein levels. The percentage of inhibition of the WST-1 reduction rate was calculated as a measure of SOD activity.

#### Real-time quantitative PCR (qRT–PCR)

qRT–PCR was performed as previously described.^39^ The total RNA level was normalized in each reaction using *β-actin* cDNA as an internal standard. The primers used are listed in below table.

**Table:** List of primer for real time q-PCR

*SOD1*	*5′-GGTGTGGCCGATGTGTCTAT-3′*	*5′-ACTTCCAGCGTTTCCTGTCT-3′*
*SOD2*	*5′-TGGCCAAGGGAGATGTTACA-3′*	*5′-CTTCCAGCAACTCCCCTTTG-3′*
*IL-6*	*5′-CCAGCTATGAACTCCTTCTC-3′*	*5′-GCTTGTTCCTCACATCTCTC-3′*
*TNF-α*	*5′-AGCCTCTCTTCTCCTTCCTGATCGT-3′*	*5′-GGCTGATTAGAGAGAGGTCCCTG-3′*
*IL-1α*	*5′-AACCAGTGCTGCTGAAGGA-3′*	*5′-TTCTTAGTGCCGTGAGTTTCC-3-3′*
*IL-1β*	*5′-CTGTCCTGCGTGTTGAAAGA-3′*	*5′-TTGGGTAATTTTTGGGATCTACA-3′*
*HO-1*	*5′-AGACTGCGTTCCTGCTCAAC-3′*	*5′-GGCTCTGGTCCTTGGTGTC-3′*
*GRP78*	*5′-TAGCGTATGGTGCTGCTGTC-3′*	*5′-TTTGTCAGGGGTCTTTCACC-3′*
*HSP47*	*5′-GACCACCCCTTCATCTTCCT-3′*	*5′-ACTCGTCTCGCATCTTGTCA-3′*
*β-actin*	*5′-GGACTTCGAGCAAGAGATGG-3′*	*5′-AGCACTGTGTTGGCGTACAG-3′*

#### Immunoblotting analysis

Immunoblotting experiments were conducted as previously described.^39^ The antibodies used for immunoblotting were specific to the following proteins: GRP78, GRP94, HSP70, Iκ-B, PDI, Sirt3, Lamin A/C, Nrf2, NF-κB(p65) and SOD2 (Cell Signaling); HSP47 and Acetyl-SOD2(K68) (Abcam); and β-actin and α-tubulin (Sigma). The antibodies were diluted at a 1:1000 ratio, except for the anti-β-actin antibody (1:10000 dilution). Secondary antibodies were purchased from Promega (anti-rabbit and anti-mouse, used at a 1:5000 dilution).

#### siRNA-mediated gene targeting

NB1RGB cells were transfected with siRNA specific for SOD2 (siSOD2; the siRNA SMARTpool for human SOD2 from Dharmacon, Lafayette, CO, USA) and controls (Santa Cruz, Santa Cruz, CA, USA) using the Lipofectamine RNAiMAX transfection reagent (Invitrogen, Carlsbad, CA, USA) according to the manufacturer’s instructions. Cells were transfected with 25 nM of siControl or siSOD2.

#### Secreted alkaline phosphatase (SEAP) assay

Cells were transduced with the pSEAP2-control vector (Takara Bio, Tokyo, Japan). Culture supernatants were then harvested and assayed for SEAP activity using the Great EscAPe SEAP Reporter System #631736 (Takara Bio, Tokyo, Japan). pRL-Renilla luciferase control vector was also measured using the Dual-Luciferase Assay system #E1910 (Promega, Madison, WI, USA). We have presented the data as percentages, which represent the RLU of each sample divided by the RLU of non-treated cells sample.

### Quantification and statistical analyses

Differences in mean values were evaluated using Student’s t-test for unpaired results to assess differences between two groups, and ANOVA (one cell type treated with more than two compounds) or two-way ANOVA (two cell types treated with more than one compound), followed by the Tukey-HSD test. A p-value of <0.05 was used to indicate statistical significance (Mac statistical analysis software, Esumi Co., Tokyo, Japan). n.s.: not significant, ∗p < 0.05, ∗∗p < 0.01.

## Data Availability

• Data reported in this paper will be shared by the lead contact upon request. • This paper does not report original code. • Any additional information required to reanalyze the data reported in this paper is available from the lead contact upon request.
