# Supplementary material for: Restoration of mitochondrial function by Spirulina polysaccharide via upregulated SOD2 in aging fibroblasts
Source: iScience. 2023 Jun 14;26(7):107113. doi: 10.1016/j.isci.2023.107113 (PMC10319841; doi:10.1016/j.isci.2023.107113)
Supplement: Document S1. Figures S1–S7 [file mmc1.pdf]

**iScience, Volume *26***

# Supplemental information

# Restoration of mitochondrial function by *Spirulina* polysaccharide via upregulated SOD2 in aging fibroblasts

**Kayo Machihara, Shoma Oki, Yuka Maejima, Sou Kageyama, Ayumu Onda, Yurino Koseki, Yasuyuki Imai, and Takushi Namba**

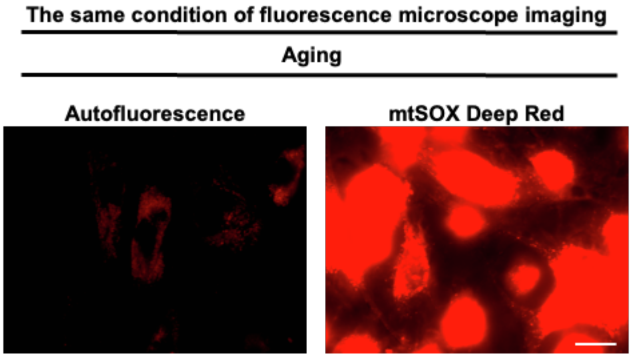


**Supplementary Figure S1. The mtSOX Deep Red fluorescence intensity is significantly higher compared to autofluorescence intensity in aging NB1RGB cells, Related to Figure 3.**

The autofluorescence (left panel) and mtSOX Deep Red fluorescence (right panel) were compared to same fluorescence microscope imaging condition in aging NB1RGB cells (scale bar, 20 μm).

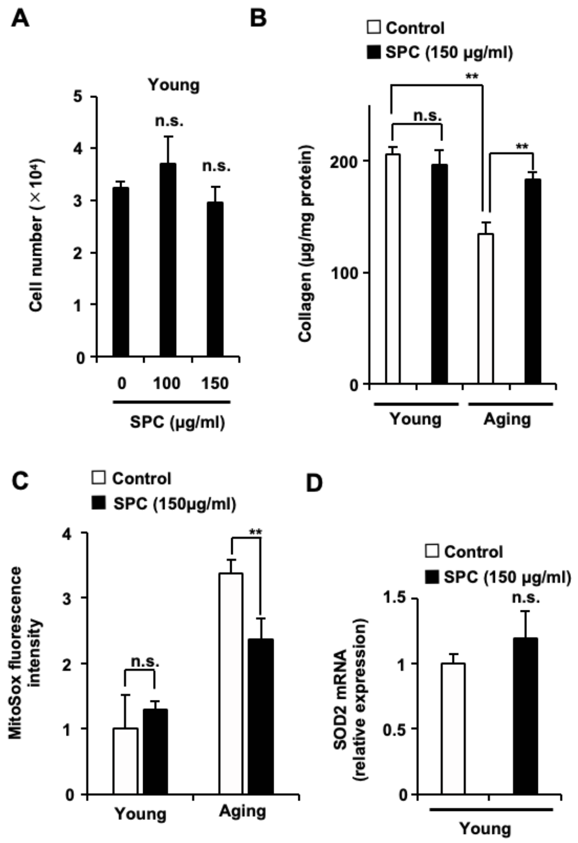


**Supplementary Figure S2. SPC did not affect collagen production and ROS production in young NB1RGB cells, Related to Figures 3 and 4.**

(A) SPC did not exhibit cell toxicity in young NB1RGB cells. Young NB1RGB cells were treated with or without indicated concentration of SPC for 48 h. Cell viability was determined using the cell count assay. (B) SPC did not stimulate collagen production in young NB1RGB cells. Young or aging NB1RGB cells were treated with or without indicated concentration of SPC for 48 h. Collagen production was normalized by total amount of protein. (C) SPC did not eliminated ROS in young NB1RGB cells. Young and aging NB1RGB cells were treated with indicated concentration of SPC for 48 h. The ROS level in the cells was determined using the MitoSOX staining assay. The fluorescence intensity of MitoSOX was determined by using plate reader. (D) SPC did not induced SOD2 mRNA expression in young NB1RGB cells. Young NB1RGB cells were treated with or without SPC at the indicated concentrations for 24 h and subjected to qPCR. Data are presented as the mean ± SD of three simultaneously performed experiments, using three wells on the same plate (C) or different plate (A, B, D). *P* values were calculated using Student's *t* test (D), ANOVA following Tukey-HSD test (A) and two-way ANOVA following Tukey-HSD test (B, C); n.s.: not significant, $^{**}P < 0.01$.

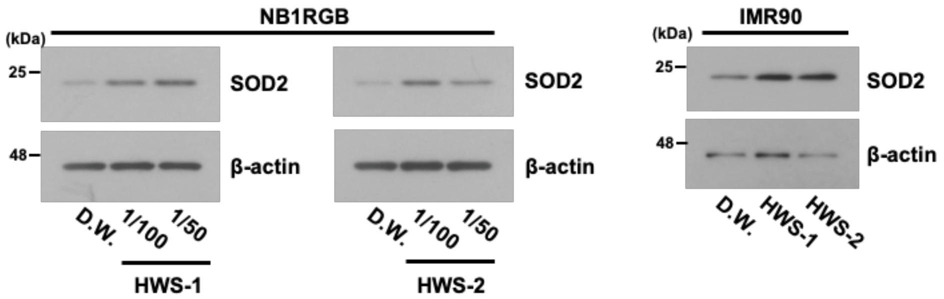


**Supplementary Figure S3. HWS stimulated SOD2 protein expression, Related to Figure 4.**

HWS induced SOD2 expression. Aging NB1RGB and IMR90 cells were treated with the indicated dilutions or 1/100 dilution of HWSs for 24 h. Cells were subjected to immunoblotting using the indicated antibodies.

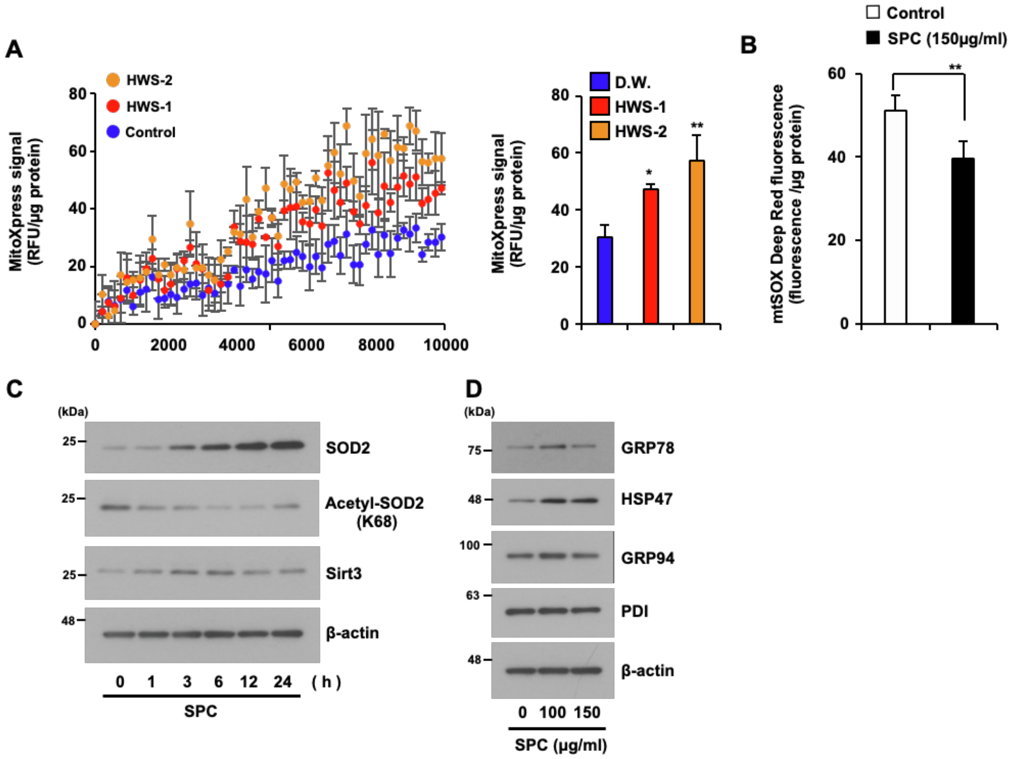


**Supplementary Figure S4. SPC and HWS stimulated mitochondrial function and SOD2 protein expression in aging IMR90 cells, Related to Figures 2, 3 and 4.**

(A) Mitochondrial oxygen consumption is stimulated by HWS-1 or HWS-2 treatment in Aging IMR90 cells. Aging IMR90 cells were treated with HWS-1 or HWS-2 for 48 hours. TR-F of the MitoXpress probe was performed using the same procedure described in Fig 1C (right panel), and the final measurement point was represented by a bar graph (right panel). (B) SPC eliminated ROS in aging IMR90 cells. Aging IMR90 cells were treated with 150 μg/ml of SPC for 48 h. The ROS level in the cells was determined using the mtSOX Deep Red staining assay. The fluorescence intensity of mtSOX Deep Red was determined after subtracting the autofluorescence intensity of the cells by using plate reader. (C, D) Aging IMR90 cells were treated with 150 μg/ml of SPC (C) or indicated concentration of SPC (D) for the indicated time periods (C) or 24 h (D). Cells were subjected to immunoblotting using the indicated antibodies. Data are presented as the mean ± SD of three simultaneously performed experiments, using three wells on the same plate (A, B). *P* values were calculated using ANOVA following Tukey-HSD test (A, B); *$P < 0.05$, **$P < 0.01$.

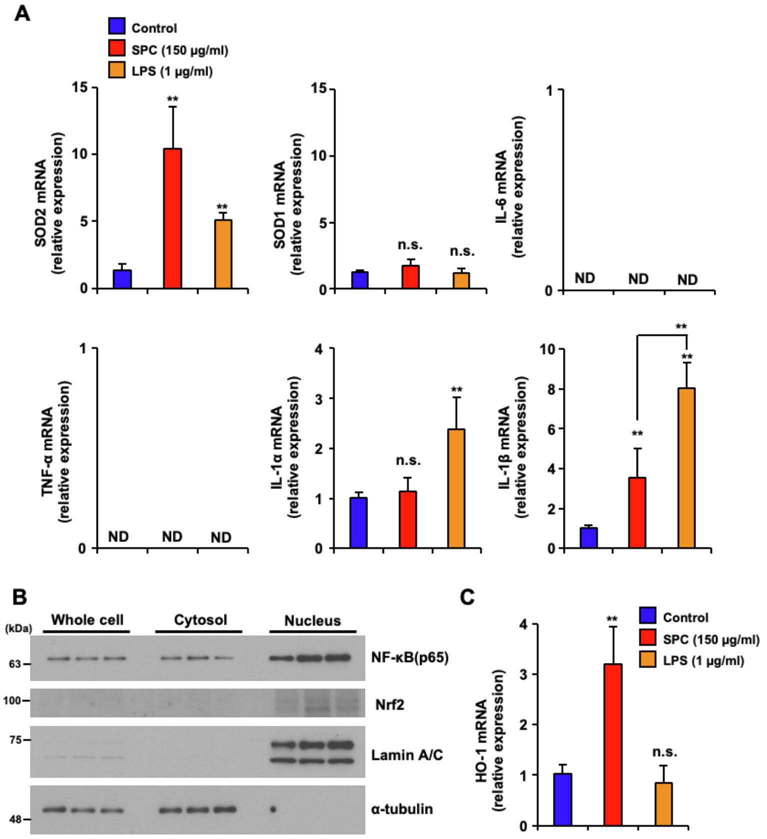


**Supplementary Figure S5. SPC did not activate inflammatory signaling in aging IMR90 cells, Related to Figures 4 and5.**

(A-C) Aging IMR90 cells were treated with SPC or LPS at the indicated concentrations for 24 h (A, C) or 4 h (B); they were then subjected to qPCR (A, C) or fractionation assay using the same procedure described in Fig 1C (B). Data are presented as the mean ± SD of three simultaneously performed experiments, using three wells on independent plates (A, C). *P* values were calculated using ANOVA following Tukey-HSD test (A, C); n.s.: not significant, **P* < 0.05, ***P* < 0.01.

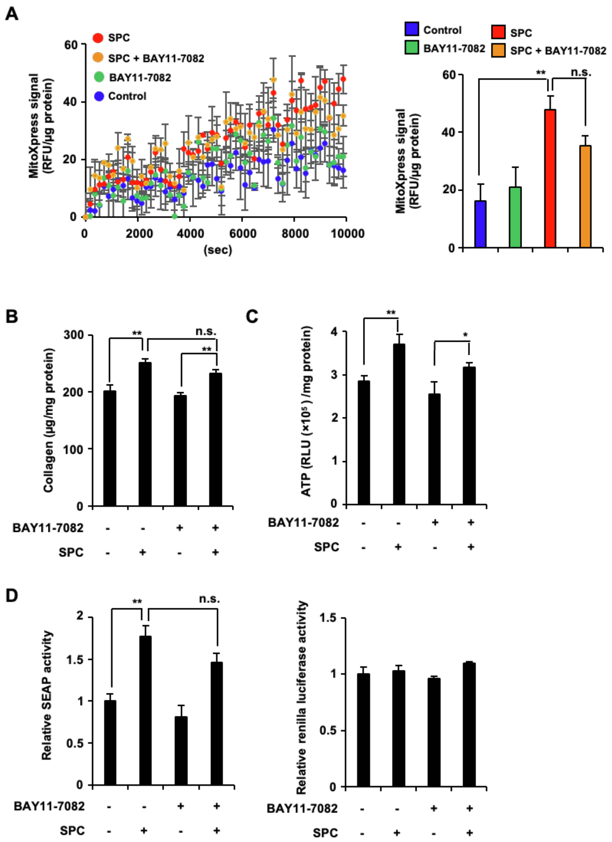


**Supplementary Figure S6. Suppression of Iκ-B degradation partially inhibited the SPC-induced upregulation of mitochondrial function, Related to Figures 4 and 6.**

(A-C) Suppression of Iκ-B degradation partially inhibited the SPC-induced upregulation of ATP, but not oxygen consumption and collagen production. Aging IMR90 cells were treated with or without 10 µM of BAY11-7082 and 150 µg/ml of SPC for 48 h (B, C) or 24 h (A); they were then subjected to MitoXpress assay (A), collagen assay (B) and ATP assay (C). (D) Aging IMR90 cells expressing SEAP were transduced with a pSEAP2-Control vector and washed 18 h after transduction; the cells were then treated with or without 150 µg/ml of SPC for 24 h. The medium was then changed, and the cells were cultured for another 12 h. Culture media were then analyzed for SEAP activity, and luminescence was normalized to cell number (left panel). Aging NB1RGB cells expressing renilla luciferase were transduced with a pRL-Renilla luciferase control vector and washed 18 h after transduction; the cells were then treated with or without 150 µg/ml of SPC for 24 h (right panel). The data as percentages represented the RLU of each sample divided by the RLU of non-treated cells sample. Data are presented as the mean ± SD of three simultaneously performed experiments, using three wells on the same plate (A, C, D) or three wells on independent plates (B). *P* values were calculated using ANOVA following Tukey-HSD test (A-D); n.s.: not significant, **P* < 0.05, ***P* < 0.01.

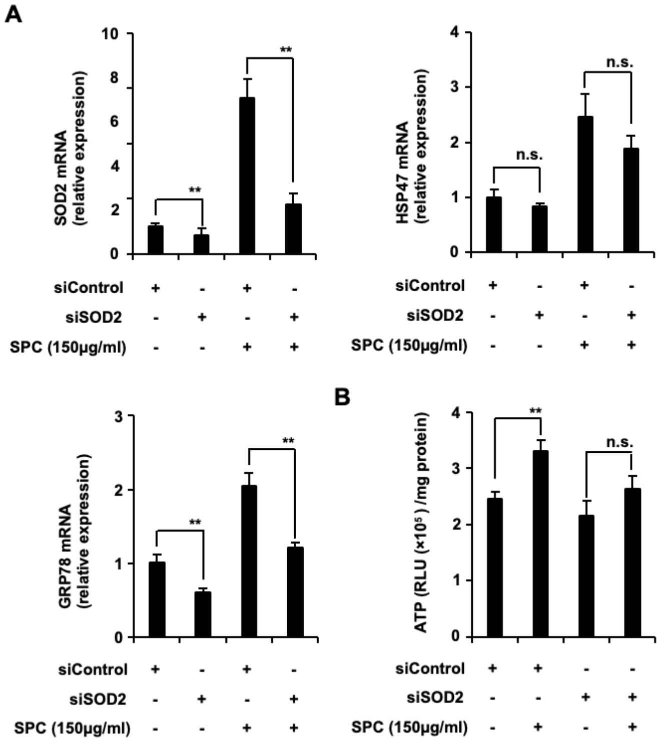


**Supplementary Figure S7. SPC-induced increase in ATP production and GRP78 expression were dependent on the upregulation of SOD2 expression in aging IMR90 cells, Related to Figure 7.**

(A, B) Aging IMR90 cells were transfected with 25 nM of siControl or siSOD2 for 24 h, followed by treatment with or without 150 μg/ml SPC for 24 h (A) or 48 h (B). Cells were subjected to qPCR (A) or ATP content (B). Data are presented as the mean ± SD of three simultaneously performed experiments, using three wells on the same plate (B) or different plate (A). *P* values were calculated using two-way ANOVA following Tukey-HSD test (A, B); n.s.: not significant, $^{**}P < 0.01$.
